# CD36 in liver diseases

**DOI:** 10.1097/HC9.0000000000000623

**Published:** 2025-01-07

**Authors:** Yi Liu, Wenwei Yin

**Affiliations:** Key Laboratory of Molecular Biology for Infectious Diseases (Ministry of Education), Department of Infectious Diseases, Institute for Viral Hepatitis, The Second Affiliated Hospital of Chongqing Medical University, Chongqing, China

**Keywords:** CD36, liver cancer, liver fibrosis, NAFLD, NASH

## Abstract

Cluster of differentiation 36 (CD36) is a transmembrane glycoprotein with the ability to bind to multiple ligands and perform diverse functions. Through the recognition of long-chain fatty acids, proteins containing thrombospondin structural homology repeat domains such as thrombospondin-1, and molecules with molecular structures consistent with danger- or pathogen-associated molecular patterns, CD36 participates in various physiological and pathological processes of the body. CD36 is widely expressed in various cell types, including hepatocytes and KCs in the liver, where it plays a pivotal role in lipid metabolism, inflammation, and oxidative stress. Accumulating evidence suggests that CD36 plays a complex role in the development of nonalcoholic simple fatty liver disease and NASH and contributes to the pathogenesis of inflammatory liver injury, hepatitis B/hepatitis C, liver fibrosis, and liver cancer. This review summarizes the current understanding of the structural properties, expression patterns, and functional mechanisms of CD36 in the context of liver pathophysiology. Furthermore, the potential of CD36 as a therapeutic target for the prevention and treatment of liver diseases is highlighted.

## Graphical abstract

**Figure:**
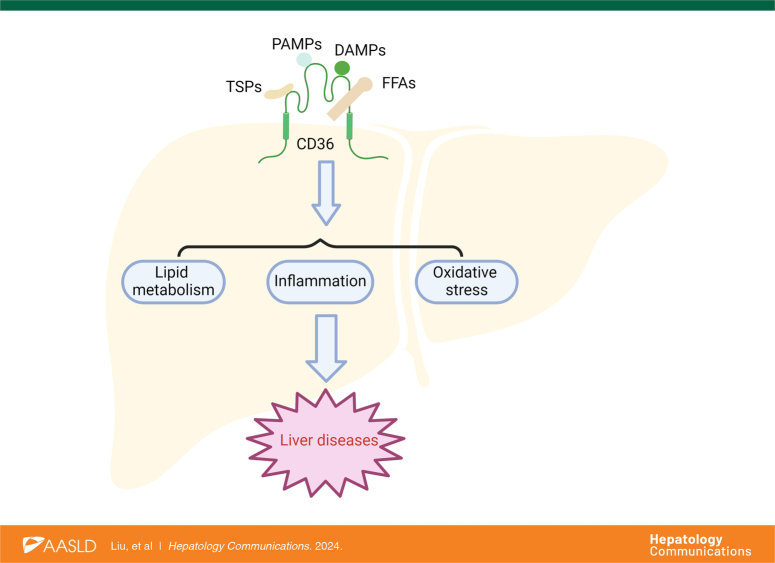

## INTRODUCTION

**FIGURE 1:**
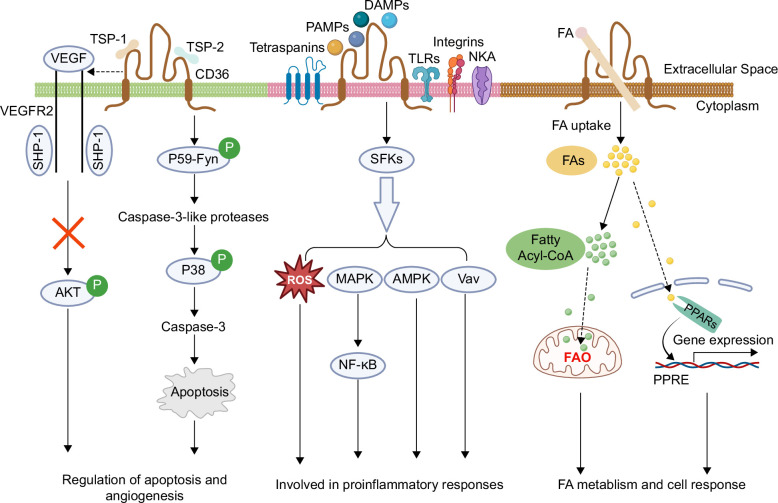
CD36 serves as both a signal transducer and a fatty acid transporter. Binding of CD36 to TSR domain proteins, including TSP-1 and -2, activates phosphorylation of Fyn, and subsequently activates p38 and caspase, inducing apoptosis and antiangiogenesis in vascular endothelial cells. TSP-1 interactions with CD36 can also recruit the phosphatase SHP1 to VEGFR2 in endothelial cells, and thereby inhibit VEGF-induced angiogenesis. In response to extracellular signals such as DAMPs and PAMPs, CD36 assembles and interacts with other membrane receptors, including NKA, TLRs, integrins, and tetraspanins, forming different signaling complexes; the signaling complex triggers an intracellular signaling cascade that starts with SFK activation and then signals downstream effectors including MAPK, AMPK, and Vav. Activation of these effectors leads to ROS production and NF-κB pathway activation, which results in proinflammatory responses. Meanwhile, CD36 could bind to LCFAs and facilitate their transmembrane transport, which impacts fatty acid metabolism and may contribute to the activation of the PPARs pathway. Created with Biorender.com. Abbreviations: AMPK, adenosine 5′monophosphate-activated protein kinase; CD36, cluster of differentiation 36; DAMP, danger-associated molecular pattern; LCFA, long-chain fatty acid; MAPK, mitogen-activated protein kinase; NKA, neurokinin A; PAMP, pathogen-associated molecular pattern; PPAR, peroxisome proliferator–activated receptor; ROS, reactive oxygen species; SFK, src-family kinase; TLR, Toll-like receptor; TSR, thrombospondin structural homology repeat.

CD36, also known as fatty acid transporter, transmembrane glycoprotein cluster of differentiation 36, or scavenger receptor class B protein, encoded by the human CD36 gene, consists of a single peptide chain of 472 amino acids and is a glycoprotein with a molecular weight of 88 kD possessing 2 transmembrane domains.^1^ The extracellular domain has a site for binding ligands, and the N- and C-termini (intracellular domain) in the cytoplasm can transduce signals into the cell, activating downstream signaling pathways.^2^ CD36 is a multi-ligand pattern recognition receptor capable of interacting with many structurally distinct ligands, falling into 3 broad categories—proteins containing thrombospondin structural homology repeat domains, long-chain fatty acids (LCFAs), and molecules exhibiting molecular structures consistent with danger- or pathogen-associated molecular patterns (Figure 1). The binding of CD36 to thrombospondin structural homology repeat domain proteins, including thrombospondin-1 and -2 (TSP-1/2), activates phosphorylation of Fyn, and subsequently activates p38 and caspase, inducing apoptosis and antiangiogenesis in vascular endothelial cells. TSP-1 interactions with CD36 can also recruit the phosphatase SHP1 to VEGF receptor2 in endothelial cells, and thereby inhibit VEGF-induced angiogenesis. As a typical pattern recognition receptor, upon binding to extracellular ligands such as danger- and pathogen-associated molecular patterns, CD36 assembles and interacts with other membrane receptors, including neurokinin A, Toll-like receptors, integrins, and tetraspanins, forming different signaling complexes; the signaling complex triggers an intracellular signaling cascade that starts with src-family kinase activation and then signals downstream effectors including mitogen-activated protein kinase, adenosine 5′monophosphate-activated protein kinase, and guanine nucleotide exchange factor Vav. Activation of these effectors leads to reactive oxygen species (ROS) production and NF-κB pathway activation, which results in proinflammatory responses.^3^ Furthermore, CD36 could bind to LCFAs and facilitate their transmembrane transport, which impacts fatty acids metabolism and may contribute to the activation of the peroxisome proliferator–activated receptors (PPARs) pathway.^4^^,^^5^ Activation of PPARs could further influence cell response, including cell proliferation, activation, and death.^5^

Under normal circumstances, CD36 is strongly expressed in tissues that primarily rely on free fatty acids (FFAs) as their main energy source, such as adipose tissue, the heart, liver, skeletal muscles, and kidneys.^6^ It mediates the absorption of FFAs and serves as an energy source for these tissues. In the liver, CD36 has a low basal expression level, but during high-fat feeding and in cases of NAFLD, the expression of CD36 significantly increases.^6^ Currently, there is a clearer understanding of the biological functions and signaling pathways of CD36, with increasing evidence pointing to its significant role in the progression of liver diseases. In the following review, we will focus on the latest advancements regarding CD36 in liver diseases and briefly discuss the potential of CD36 as a therapeutic target for liver diseases.

## CD36 AND NAFLD

NAFLD is a metabolic liver disease characterized by the accumulation of a significant amount of fat within liver cells. It has emerged as a leading cause of chronic liver disease, with a global prevalence exceeding 25% and showing an increasing trend year by year.^7^ Simple fatty liver (hepatic steatosis) represents an early stage of NAFLD, characterized by the accumulation of fat droplets in liver cells exceeding 5%, primarily composed of triglycerides.^8^ It is known that CD36 enhances the uptake of FFAs by the liver, and the liver toxicity caused by an excessive influx of FFAs into liver cells is a key factor in NAFLD.^9^

Several clinical studies have demonstrated increased liver CD36 expression levels in NAFLD.^9^^–^^11^ The expression of CD36 is not only significantly upregulated in patients with fatty liver but also positively correlates with the liver’s fat content in these patients.^10^^,^^12^ At the mRNA and protein levels, CD36 was also upregulated in patients with NAFLD and was significantly related to the extent of fatty liver.^13^ Limited studies suggest that soluble CD36 may serve as a novel potential biomarker for NAFLD.^9^^,^^14^ Animal models induced by obesity and a high-fat diet (HFD) also demonstrate an elevation in CD36 levels in hepatic steatosis. CD36 was increased 20 times and 6 times at the mRNA and protein levels, respectively, in the livers of ob/ob mice compared to wild-type mice.^15^ In addition, compared with low-fat diet mice, the content of triglyceride in mice fed an HFD increased 1.7 times, and the expression of CD36 at the protein level increased by 2.6 times.^6^ Thus, hepatic CD36 expression is closely associated with the development of NAFLD.

CD36, as a fatty acid translocase, plays a vital function in the uptake of FAAs, and the mechanisms have been elucidated by some studies. At the sarcolemmal lipid raft site of cardiac and skeletal muscle, CD36 promotes fatty acid entry into cells through interaction with plasma membrane fatty acid binding protein and cytosolic fatty acid binding protein.^16^ In short-term fatty acid uptake, the regulation of the rate of fatty acid uptake by CD36 is influenced by muscle contraction signals and insulin levels, while long-term regulation is promoted by activation of peroxisome proliferation–activated receptors to promote transcription of the CD36 gene.^16^ In adipose tissue, CD36 not only mediates fatty acid uptake but is also involved in modulating the process of lipolysis and fatty acid reesterification,^17^^,^^18^ as well as in the correlation with hepatic clock stabilization and lipid homeostasis in liver cells.^19^^,^^20^ Similarly, CD36 promotes the uptake of fatty acids by liver cells. Upon high insulin concentration environment (80 mU/L), CD36 is translocated to the plasma membrane of obese Zucker mouse hepatocytes in increased numbers at the mRNA and protein levels, thus potentiating fatty acid uptake and the content of triglyceride synthesis.^21^ In human hepatoma cells, CD36 overexpression significantly increased fatty acid uptake.^22^ Moreover, transfection of primary hepatocytes with recombinant adenovirus overexpressing CD36 also results in lipid accumulation.^6^ Zhou et al^23^ and Zeng et al^24^ reported that obesity-induced upregulation of the nuclear orphan receptor NR2F6, as well as INSIG2-dependent SREBP1 and the transcription factors ATF4 and SOX2, promotes CD36 expression in liver tissues, leading to enhanced fatty acid uptake and the development of NAFLD.

Animal models with CD36 gene deletion (CD36^−/−^ mice) have provided insights into the function of CD36 in NAFLD. Coburn et al demonstrated that compared with wild-type mice, the fatty acid uptake rate in CD36^−/−^ mice was reduced by 50%–80%, 40%–75%, and 60%–70% in the heart, skeletal muscle, and adipose tissue, respectively.^25^ Surprisingly, CD36 deletion resulted in an unexpected increase in hepatic triglycerides and hepatic lipid accumulation, which was associated with upregulation of genes and proteins of de novo lipogenesis and decreased liver triglyceride secretion.^26^^,^^27^ Several CD36-related studies have indicated that liver steatosis is more severe in CD36^−/−^ mice. Vroegrijk et al^28^ proposed that CD36^−/−^ mouse liver steatosis is aggravated due to lower fatty acid uptake in muscle and adipose tissue, increased lipolysis in adipose tissue, and higher fatty acid flow to the liver. In ob/ob mice, systemic CD36 deficiency upregulates the level of prostaglandins and then inhibits the secretion of liver VLDL, thereby promoting liver steatosis.^15^ In humans, CD36 deficiency, which is relatively common (2%–7%) in persons of Asian and African descent, has been reported to exhibit insulin resistance, atherosclerotic heart disease, hyperlipidemia, and a propensity to develop NAFLD and atherosclerotic heart disease.^29^^,^^30^

To avoid the effects of whole-body knockout of CD36, Wilson et al^31^ established a CD36 hepatocyte-specific deletion mouse model and proved that CD36 deletion in hepatocytes reduces liver steatosis and improves liver inflammation markers. With hepatocyte-specific CD36 knockout mice, Zeng et al^24^ also identified hepatocyte CD36 as a key regulator for de novo lipogenesis in the liver. As such, CD36 may be involved in the development of NAFLD not only by promoting fatty acid uptake but also by upregulating initio lipogenesis in the liver. Despite the well-documented function of CD36 in fatty acid uptake and the close association of CD36 with the development of NAFLD, hepatic overexpression of CD36 unexpectedly attenuates HFD-induced hepatic steatosis and insulin resistance.^32^

Overall, CD36 expression in the liver increased significantly in both patients with NAFLD and mouse models; in parallel with CD36 expression, fatty acid uptake increased significantly, indicating that CD36 is closely related to the development of fatty liver. However, multiple studies have shown that CD36 deficiency paradoxically promotes the progression of NAFLD. CD36 is widely expressed in multiple cell types in the liver; while hepatocyte-specific CD36 deletion was protected against HFD-induced hepatic steatosis, the deletion of CD36 in endothelial cells unexpectedly increased liver triglyceride content, possibly due to altered vascular permeability or changes in lipid transport.^31^^,^^33^ These findings indicated that the role of CD36 in NAFLD may be cell-type dependent. Although mice lacking CD36 exhibit increased plasma FFA and triglyceride levels when administered an HFD, CD36 deficiency displayed a slight effect on lipid metabolism under normal chow diet conditions, suggesting that different dietary states may change the expression of CD36 and the associated metabolic phenotype.^27^^,^^34^ Further experimental research is needed to better understand the role of CD36 in regulating hepatic lipid homeostasis and its impact on the progression of hepatosteatosis to NAFLD. Understanding these complexities will be crucial for developing targeted therapeutic strategies for NAFLD.

## CD36 AND NASH

**FIGURE 2:**
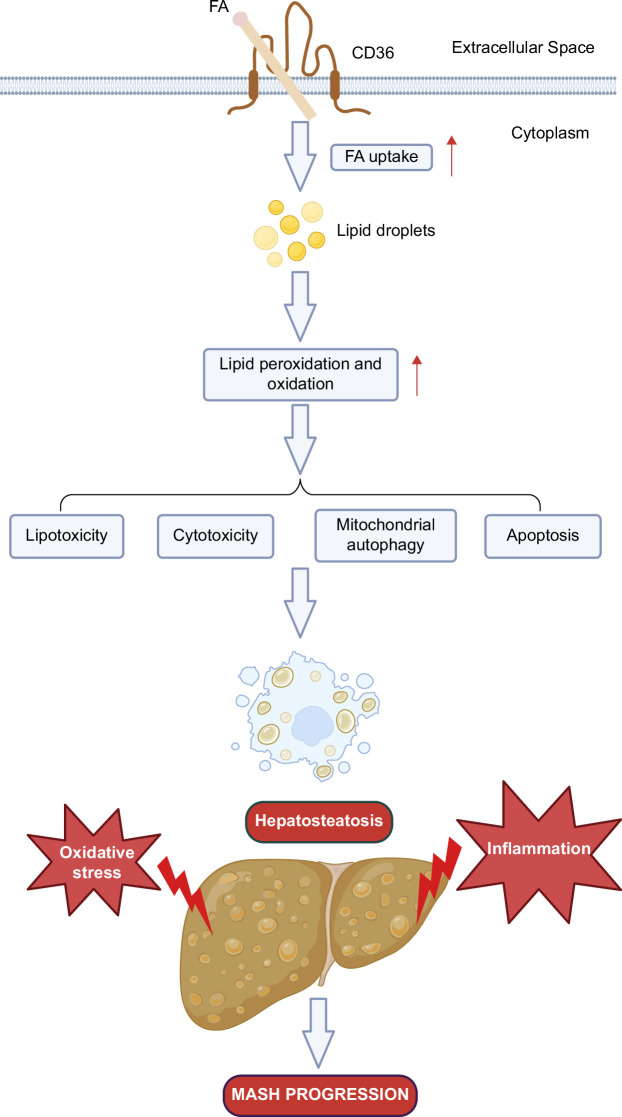
CD36-mediated excessive uptake of fatty acids contributes to NASH. CD36 increases FFA uptake in the liver, and excessive FFA influx could lead to peroxidation and oxidative stress in hepatocytes, causing lipid disorders. Lipid disorders, in turn, induce lipotoxicity, cytotoxicity, mitochondrial autophagy, and apoptosis in hepatocytes, promoting tissue damage and inflammation, which drives hepatosteatosis onset and might contribute to its progression to NASH. Created with Biorender.com. Abbreviations: CD36, cluster of differentiation 36; FFA, free fatty acid.

NASH is characterized by massive lipid accumulation in hepatocytes accompanied by liver tissue inflammation and damage. Different from simple steatosis, which is considered reversible, NASH may develop into fibrosis, cirrhosis, or even HCC, a more severe stage in NAFLD.^35^ It is now recognized that CD36 increases FFA uptake in the liver and excessive FFA influx could lead to peroxidation and oxidative stress in hepatocytes, causing lipid disorders. Lipid disorders in turn induce lipotoxicity, cytotoxicity, mitochondrial autophagy, and apoptosis in hepatocytes, promoting tissue damage and inflammation, which drives hepatosteatosis onset and might contribute to its progression to NASH (Figure 2).^9^

The 2-hit hypothesis suggests that hepatic steatosis functions as the first hit, and additional oxidative stress functions as the second hit, which is a key step, contributing to the development of simple steatosis to NASH.^36^ The study by Yimin et al^37^ demonstrated that elevated CD36 expression in hepatocytes and hepatic macrophages during NASH development plays a critical role in mediating the associated pathophysiological processes. Bieghs et al^34^ confirmed the involvement of scavenger receptor-A and CD36 in KCs in the early formation and progression of NASH. The team further demonstrated that KC CD36 phagocytosed modified lipids (eg, oxidized low-density lipoprotein, oxLDL), resulting in cholesterol accumulation in lysosomes and causing liver inflammation, thereby elevating the risk of NASH.^38^ In addition, Bechmann et al^39^ suggest that activated CD36 function may result in hepatocyte apoptosis, which is also considered an important event in NASH.

It has been confirmed that human CD36 possesses 4 palmitoylation sites, equally distributed at the N-terminus and C-terminus, occupying the cysteines 3, 7, 464, and 466, respectively.^40^ Recently, the report by Zhao and colleagues demonstrated that palmitoylation of CD36 plays an extremely important role in the progression of NASH. Blocking the palmitoylation of CD36 not only reduces fatty acid uptake in hepatocytes by suppressing the formation of the CD36/Fyn/Lyn complex and promotes β-oxidation of fatty acids but also alleviates liver inflammation by inhibiting the C-Jun N-terminal kinase signaling pathway.^41^ Inhibition of CD36 palmitoylation could also enhance hepatic fatty acid β-oxidation by promoting CD36 localization to the mitochondria of hepatocytes.^41^ Hence, CD36 palmitoylation appears crucial in the pathogenesis of NASH, and inhibiting CD36 palmitoylation might represent a significant therapeutic strategy for treating NASH.

Compared with wild-type mice, deletion of CD36 causes a decrease in the level of ROS in the liver, with decreased expression of histone deacetylase 2 and elevated activation of acetyl histone 3, which binds to the monocyte chemotactic protein-1 promoter, leading to the activation of monocyte chemotactic protein-1 transcription and promoting NASH.^27^

Combined with the above findings, CD36 acts far more than transport fatty acids. In addition to binding to modified lipids triggering intracellular inflammation, the function of CD36 also involves the activation of associated inflammatory metabolic signaling pathways and induction of apoptosis, playing an indispensable role in the occurrence and development of NASH.

## CD36 AND LIVER INJURY

Currently, a series of studies conducted on both humans and mice indicate the involvement of CD36 in the pathological processes of liver injury. Reports suggest that in patients with impaired glucose tolerance, the circulating levels of sCD36, the noncellular soluble form of CD36 in plasma, are positively correlated with serum ALT, AST, and GGT, whereas there is no such correlation in patients with normal glucose tolerance.^42^ This association suggests that circulating sCD36 might serve as a potential new marker for liver injury in patients with impaired glucose tolerance.^42^^,^^43^ Proprotein convertase subtilisin/kexin type 9 (PCSK9) is a serine protease that plays a crucial role in lipid metabolism. It is primarily expressed in the liver and is known to reduce the abundance of receptors on the surface of liver cells that are responsible for absorbing lipids from circulation.^44^ PCSK9 can protect mice fed an HDL diet against hepatic steatosis and liver injury by inhibiting CD36 expression and fatty acid uptake.^45^^,^^46^ The calcium-independent phospholipase A2β (iPla2β) is an enzyme that belongs to the phospholipase A2 family. It catalyzes the hydrolysis of glycerophospholipids, releasing FFAs and lysophospholipids.^47^ Hepatocyte-specific deletion of iPla2β can alleviate hepatocellular damage in methionine and choline-deficient diet mice by suppressing the expression of CD36 and other lipid uptake–related proteins.^22^^,^^47^^,^^48^

Previous research reported that CD36 can form a complex with the Toll-like receptors 4/6 after binding with its ligand oxLDL, further activating the NF-κB pathway and triggering inflammatory responses.^49^ Multiple follow-up studies have suggested that the inflammatory response triggered by CD36 signaling plays an important role in liver injury. For instance, sirtuin 1 upregulation attenuates liver injury by inhibiting CD36 expression and the NF-κB signaling pathway in HFD-induced mice.^50^ Notably, CD36 exerts a significant proinflammatory role in concanavalin A–induced liver injury.^51^ Deletion of CD36 alleviates concanavalin A–induced liver injury by reducing hepatic inflammation and hepatocyte apoptosis, and using the CD36 inhibitor genistein to block CD36-Lyn signaling can reduce liver injury caused by concanavalin A.^51^ Excessive acetaminophen intake can lead to liver toxicity and even acute liver failure. Recent studies suggest that sterile inflammation may play a crucial role in acetaminophen-induced liver damage.^52^ CD36 has been positioned as a central regulator of sterile inflammation by coordinating NACHT, LRR, and PYD domains–containing protein 3 inflammasome activation.^53^ Following acetaminophen intake, an increase in CD36 expression occurs, and CD36 deficiency could ameliorate acetaminophen-induced acute liver injury and inflammatory responses by decreasing C-Jun N-terminal kinase activation.^54^

Together, these results indicate that CD36 induces lipid deposition and mediates the activation of inflammatory pathways, resulting in a liver inflammatory state and contributing to liver injury. As a mediator of liver injury, targeting CD36 may help to identify novel therapeutic targets for the treatment of this disease. However, there are some contrary findings. Recent studies have shown that CD36 is involved in the activation of the autophagy program in macrophages and that autophagy activation by the CD5L/CD36/ATG7 pathway plays a protective role in hepatic ischemia/reperfusion injury.^55^^,^^56^ Notch signaling in hepatocytes is normally inactive, and its activation is positively associated with a higher serum ALT level and a higher fibrosis score in patients with NAFLD.^57^ Notch1 Intracellular Domain (N1ICD) is a key signaling molecule involved in the Notch pathway, which plays a crucial role in cell fate determination and inflammation. Recent animal experiments indicate that CD36 in hepatocytes can exert a protective effect against liver fibrosis and injury by blocking the production of N1ICD.^58^ Specifically, CD36 anchors Notch1 in lipid rafts and blocks the Notch1/γ-secretase interaction, inhibiting γ-secretase–mediated production of N1ICD in hepatocytes and thus exerting a protective effect against NASH diet–induced liver injury and fibrosis.^58^ Therefore, the role of CD36 in liver damage induced by different factors is complex and further research is needed to clarify it.

## CD36 AND HEPATITIS B/HEPATITIS C

Acute and chronic hepatitis caused by HBV and HCV infection has become a global health problem. Patients with hepatitis B or C are at an increased risk of liver decompensation, cirrhosis, and HCC.^59^

CD36 is considered a critical regulator for HIV in completing virus transcription, assembly, storage, and release cycles.^60^^,^^61^ In HepG2.2.15 cells, which stably replicate HBV and express viral-related proteins, there is a significant increase in CD36 expression, and overexpression of CD36 promotes HBV replication.^62^ A subsequent study conducted in HepG2.2.15 and HepAD38 cells revealed that the molecular mechanism by which CD36 overexpression promotes HBV replication is by increasing intracellular calcium levels and promoting Src kinase phosphorylation.^63^ Evidence suggests that circulating soluble CD36 serves as a novel noninvasive biomarker predicting liver failure and prognosis in chronic HBV-infected patients.^64^ Interestingly, a study involving patients with chronic hepatitis B found increased expression of CD36 in peripheral blood monocytes when patients were anxious.^65^ Administration of tenofovir-disoproxil-fumarate could modulate lipid metabolism by upregulating hepatic CD36 through PPAR-α activation in patients with chronic hepatitis B.^66^

CD36 has also been proposed to be involved in HCV infection. Multiple clinical studies have shown increased expression of CD36 in the liver of patients with chronic hepatitis C.^13^^,^^67^ Cheng et al^68^ proved that the stages of HCV attachment and entry into host cells are associated with CD36, a mechanism linked to the interaction of CD36 and HCV E1 proteins and blocking CD36 in vitro could significantly reduce HCV replication. Another study showed that FFA released by lipoprotein lipase inhibits HCV infection, and CD36 is involved in the regulation of HCV infection by transporting FFA.^69^ It is worth noting that in addition to the increased level of CD36 in the liver, the level of sCD36 in the blood of patients with HCV infection has also been shown to be elevated.^67^^,^^70^ Meanwhile, sCD36 levels tended to decrease after antiviral treatment in some of the patients with chronic HCV infection.^67^

These findings collectively indicate the involvement of CD36 in the life cycle of both HBV and HCV. As a necessary host factor for viral infection, CD36 might serve as a potential target for antiviral therapy.

## CD36 AND LIVER FIBROSIS

Fibrosis is a common outcome of all chronic liver injuries, and prolonged liver damage may lead to the development of cirrhosis. While various cell types are involved in the process of liver fibrosis, HSCs are the primary cells responsible.^71^ HSCs, upon activation, transdifferentiate into myofibroblasts and are responsible for depositing type I collagen, thereby playing a crucial role in the pathological processes of liver fibrosis and cirrhosis.^72^

CD36 has been reported to be expressed in HSCs, vascular endothelial cells, and cholangiocytes.^34^^,^^73^^,^^74^ CD36 polymorphism (rs1761667) is associated with higher fat intake and more instances of advanced liver fibrosis in chronic HCV infection.^75^ Recently, the important profibrotic functions of CD36 signaling in activating HSCs have been demonstrated by several studies. For example, the extracellular matrix can be produced when CD36 binds to a low concentration of oxLDL, and this process can be blocked by the monoclonal antibody OKM5, known to block CD36-mediated oxLDL uptake, resulting in a 56% reduction in collagen type I synthesis in human HSCs stimulated by oxLDL.^73^ Moreover, animal experiments have identified that the MEK1/2-pERK1/2 pathway can be activated downstream of CD36 signaling, resulting in collagen-I deposition in HSCs.^76^

Other studies have also confirmed the important profibrotic functions of CD36. MiR-29a could target CD36 to ameliorate HFD-induced steatohepatitis and liver fibrosis in mice.^77^ Glycosylated CD36 was shown to aid the intestine in absorbing LCFAs more quickly in patients with cirrhosis and portal hypertension.^78^ CD36 can mediate oxidative stress in hepatocytes, and LCFAs induce hepatocyte activation through the oxidative stress pathway, which may be an important mechanism in the process of liver fibrosis.^79^ In conclusion, CD36 might play a key role in promoting liver fibrosis, and targeting CD36 could be a potential strategy to halt the progression of liver fibrosis.

## CD36 AND LIVER CANCER

Rapid proliferation and development of tumor cells require a significant amount of lipid uptake and metabolism.^80^ Given the crucial role of CD36 in lipid metabolism, it is reasonable to speculate that CD36 is involved in the growth, invasion, and metastasis of tumors. It has been reported that CD36 is essential for metastasis initiation in oral carcinoma, melanoma, breast cancer, and ovarian cancer, and the expression of CD36 is intimately linked to metastatic initiation and progression.^81^ Blocking CD36 has an effect on antitumor metastasis; besides, the presence of CD36+ metastasis initiation cells indicates a poor prognosis of multiple tumors in clinical patients.^81^ In addition, CD36 has been demonstrated to be enriched in cancer stem cells and coexpressed with the cancer stem cell markers integrin α6 and CD133 to drive glioblastoma progression.^82^

CD36 is highly expressed in human HCC and elevated CD36 expression exerts a stimulatory effect on HCC growth and metastasis.^83^ Pretreatment of HCC cells with PI3K/AKT/mTOR inhibitors significantly blocks the tumor-promoting effects of CD36.^83^ Elevated levels of FFAs in the plasma of patients with HCC have been observed in the liver.^84^ Nath et al^85^ further demonstrated that elevated FFA uptake through CD36/fatty acid translocase activates the Wnt and TGF-β signaling pathways, which may act as upstream activators to activate the epithelial-mesenchymal transition program to promote the progression of HCC.^85^ A study of NAFLD-associated HCC indicated that CD36-mediated oxLDL uptake induces its carcinogenic signaling.^86^ Li et al^87^ showed that CD36 activation by cartilage oligomeric matrix protein caused aberrant phosphorylation of ERK and AKT and subsequently promoted HCC growth and metastasis. CD36-mediated ferroptosis inhibits and impairs the antitumor function of CD8 T cells within the tumor and promotes the development of HCC.^88^ CD36 in cancer-associated fibroblasts provides an immune-suppressive microenvironment by inhibiting migration factors, thus promoting the progression of HCC.^89^ siRNA knockout of PCSK9 resulted in excessive CD36 expression and promoted HCC progression.^90^

The liver is the most common site of cancer metastases, and liver metastasis is highly aggressive and treatment-refractory. A recent report by Yang et al^91^ found that metastasis-associated macrophages in the liver rely on CD36 to engulf tumor cell–derived LCFAs, which drives the metabolic and functional reprogramming of macrophages and promotes liver metastasis of multiple cancers.^91^ Similarly, Pfeiler et al^92^ showed that liver macrophage CD36 crucially promoted the engulfment of tumor microvesicles and controlled the extravasation of tumor microvesicles as well as premetastatic foci formation during liver metastasis of pancreatic cancer. Taken together, these findings suggest that CD36 plays an important role in promoting HCC progression and liver metastasis of multiple malignancies and targeting CD36 may be an appealing therapeutic strategy against HCC and liver metastasis.

## INTERCELLULAR AND INTERORGAN CROSSTALK THROUGH CD36 IN LIVER DISEASES

In the context of liver diseases, CD36 has been shown to participate in both intercellular and interorgan crosstalk, contributing to the progression of conditions such as fatty liver disease, hepatitis, and fibrosis. CD36-mediated uptake of LCFAs and oxLDL by hepatocytes leads to the production of ROS and proinflammatory cytokines. These cytokines activate KCs, which in turn release more cytokines, creating a cycle of inflammation and injury.^93^ Activation of KCs can also enhance the expression of CD36 on hepatocytes, further promoting lipid uptake and ROS production.^93^ Proinflammatory cytokines, such as TNF-α and IL-6, released by hepatocytes can also act on LSECs, increasing their permeability and promoting the transmigration of immune cells into the liver, thereby further amplifying the inflammatory response.^94^ ROS produced by hepatocytes due to excessive lipid accumulation can diffuse to neighboring HSCs, activating them and promoting collagen deposition.^79^ Activated HSCs then produce more ROS, creating a positive feedback loop that amplifies liver damage and fibrosis.^95^

CD36 is involved in the bidirectional communication between the liver and adipose tissue. Adipose tissue–derived factors, such as adiponectin and leptin, can modulate CD36 expression and activity in hepatocytes.^96^ Conversely, liver-derived factors, such as cytokines and chemokines, can affect adipose tissue function and contribute to systemic metabolic dysregulation.^97^ The gut-liver axis is a critical pathway in liver disease. CD36 can mediate the uptake of dietary lipids and bacterial products from the gut into enterocytes and subsequently into the liver.^98^ This can lead to increased lipid accumulation and inflammation in the liver. In addition, CD36 on intestinal epithelial cells can influence gut barrier function and the translocation of bacteria and their products, further contributing to liver disease. CD36 is also expressed in pancreatic beta cells and can influence insulin secretion.^99^ In the context of liver disease, altered lipid metabolism and inflammation can affect pancreatic function, leading to insulin resistance and diabetes.^100^ CD36-mediated lipid uptake and signaling in the liver can thus have systemic effects on glucose homeostasis.^20^

Understanding the role of CD36 in intercellular and interorgan crosstalk provides potential targets for therapeutic intervention in liver diseases. Modulating CD36 expression or activity could help reduce lipid accumulation, inflammation, and fibrosis in the liver. In addition, targeting the gut-liver axis or the liver-adipose tissue axis through CD36 could offer new strategies for managing metabolic disorders associated with liver disease.

## CD36 AND THE TREATMENT OF LIVER DISEASE

From the above discussion, it is evident that CD36 plays a crucial role in the progression of liver diseases (summarized in Table 1). Various factors have been shown to target CD36 and influence its function, potentially playing a role in the treatment of different liver conditions (summarized in Table 2).

**TABLE 1:** The summary of the mechanisms by which CD36 affects liver disease

Disease	CD36 function	CD36^−/−^
NAFLD	(1) Liver CD36 expression is increased in NAFLD,^10^^,^^11^ (2) mediates fatty acid uptake,^16^^,^^21^ and (3) modulates the process of lipolysis and fatty acid reesterification.^17^^,^^18^	(1) The fatty acid uptake rate in CD36^−/−^ mice is reduced,^25^ (2) results in an unexpected increase in hepatic triglycerides and hepatic lipid accumulation,^26^^,^^27^ (3) promotes liver steatosis,^28^ and (4) CD36 deletion in hepatocytes attenuates fatty liver.^31^
NASH	(1) CD36 expression in hepatocytes and hepatic macrophages is increased in NASH,^37^ (2) results in hepatocyte apoptosis,^39^ and (3) CD36 palmitoylation enhances hepatic fatty acid uptake and β-oxidation.^40^^,^^41^	(1) Aggravates macrophage infiltration and hepatic inflammation^27^ and (2) promotes NASH.^27^
Liver injury	(1) sCD36 might serve as a potential new marker for liver injury,^43^ (2) induces lipid deposition and triggers inflammatory responses,^46^ and (3) promotes hepatocyte apoptosis.^48^	(1) Alleviates ConA or APAP-induced liver injury^51^^,^^54^ and (2) susceptible to NASH diet–induced liver injury.^58^
HepatitisB/Hepatitis C	(1) CD36 expression is increased by HBV and HCV,^62^^,^^63^ (2) overexpression of CD36 promotes HBV replication,^62^ and (3) involved in HCV attachment and entry into host cells.^68^	(1) Blocking CD36 in vitro could reduce HCV replication.^68^
Liver fibrosis	(1) Profibrotic functions of CD36 signaling in activating HSCs,^73^ (2) regulates hepatic EMT process,^77^ and (3) mediate oxidative stress in hepatocytes.^79^	(1) MiR-29a could alleviate liver fibrosis by repressing CD36.^77^
Liver cancer	(1) Promotes HCC progression,^83^ (2) promotes EMT in HCCs,^85^ and (3) provides immunosuppressive microenvironment for HCCs.^89^	(1) Deletion of CD36 in MAMs attenuates liver metastasis.^91^

Abbreviations: CD36, cluster of differentiation 36; EMT, epithelial-mesenchymal transition; MAM, metastasis-associated macrophage.

**TABLE 2:** The summary of CD36 as a target for the treatment of liver diseases

Disease	Substances /factor	Function
NAFLD	Firmicutes^101^	CD36 expression ↑, NAFLD risk ↑
	Early life stress^102^	CD36 expression ↑, NAFLD risk ↑
	Intermittent hypoxia (IH)^103^	CD36 expression ↓, NAFLD risk ↑
	AB-kefir^104^	CD36 expression ↓, NAFLD risk ↓
	^Capsaicin^ ^105^	CD36 expression ↑, NAFLD risk ↓
	BRL37344 (β3-adrenergic receptor agonist)^106^	CD36 expression ↓, NAFLD risk ↓
	FGF21^107^	CD36 expression ↓, NAFLD risk ↓
	Desulfovibrio vulgaris^108^	CD36 expression ↓, NAFLD risk ↓
	Diosgenin^109^	CD36 expression ↓, NAFLD risk ↓
	Naringin^110^	CD36 expression ↓, treating NAFLD
	C-Maf-inducing protein (Cmip)^111^	Modulation of CD36 pathway, treating NAFLD
	Phospholipase D1 (PLD1)^112^	CD36 expression ↓, NAFLD risk ↓
NASH	Palmitoylethanolamide (PEA)^113^	CD36 expression ↓, NASH risk ↓
	Alanyl-glutamine (Ala-Glu)^114^	CD36 expression ↓, NASH risk ↓
	Beta-patchoulene (β-PAE)^115^	CD36 expression ↓, NASH risk ↓
	METTL3^116^	CD36 expression ↓, NASH risk ↓
	Alisol B^117^	RARα-PPARr-CD36 ↓, NASH risk ↓
	GW9662^118^	Inhibition of CD36 pathway, treating NASH
Hepatitis B/Hepatitis C	Ulfo-N-succinimidyl oleate (SSO)^68^	Antiviral therapy
	Lipoprotein lipase (LPL)^69^	Antiviral therapy
Liver fibrosis	Alanyl-glutamine^114^	CD36 expression ↓, Liver Fibrosis ↓
	BAR50^119^	CD36 expression ↓, Liver Fibrosis ↓
	Galectin-3 targeting drugs^120^	CD36 expression ↓, Liver Fibrosis ↓
Liver cancer	Atg5^121^	Therapeutic targets for HCC and liver metastases
	Targeting the Nogo-B pathway^86^	Therapeutic targets for HCC and liver metastases
	HSCs-derived COMP^87^	Therapeutic targets for HCC and liver metastases
	TRAF3^122^	CD36 expression ↓, inhibiting tumor growth and metastasis

In the context of NAFLD, it has been reported that substances/factors such as Firmicutes and early life stress increased the risk of NAFLD by upregulating CD36 expression.^101^^,^^102^ In the study by Ji et al,^103^ although intermittent hypoxia increased the risk of NAFLD, the CD36 expression was decreased. In contrast, substances such as naringin, AB-kefir, capsaicin, BRL37344 (β3-adrenergic receptor agonist), diosgenin, desulfovibrio vulgaris, and FGF21 caused a decreased risk of NAFLD; and with the exception of capsaicin which increases CD36 expression, reduction in the risk of NAFLD by other drugs or factors is accompanied by a decrease in CD36 expression.^104^^–^^110^ It has been demonstrated that C-Maf–inducing protein can reversibly regulate the CD36 pathway and reduce CD36 expression, which can be used as a target for the treatment of NAFLD.^111^ Inhibition of phospholipase D1 has been implicated in the protection of NAFLD by inhibiting the CD36 pathway.^112^ Medication and physical therapy modalities mentioned above may provide new treatment strategies in the future therapy of NAFLD.

For the treatment of NASH, drugs targeting CD36 show promise. Animal experiments have suggested that alanyl-glutamine, palmitoylethanolamide, beta-patchoulene, and methyltransferase 3 may reduce the risk of NASH by decreasing CD36 expression.^113^^–^^116^ Specifically, Alisol B can lower NASH risk by weakening the RARα-PPARr-CD36 cascade.^117^ GWg9662, a PPAR antagonist, also represents a potential medication for treating NASH by inhibiting the CD36 pathway.^118^

In the context of hepatitis C, it has been observed that sulfosuccinimidyl oleate, a CD36 inhibitor, effectively inhibits HCV in mice, offering a novel direction for clinical HCV treatment.^68^ Another animal study suggests that FFAs released by lipoprotein lipase inhibit HCV infection, and CD36 participates in regulating HCV infection by transporting FFAs.^69^ This presents a new avenue for clinical HCV treatment. No treatment targeting CD36 has been identified yet in hepatitis B research.

As liver disease progresses, liver fibrosis often occurs, and the aforementioned studies highlight the significant role of CD36 in the progression of liver fibrosis. Relevant experiments indicate that Ala-Glu, BAR50, and Galectin-3–targeted drugs can all reduce CD36 expression in the liver, aiming to alleviate or even reverse liver fibrosis.^114^^,^^119^^,^^120^ This offers new therapeutic strategies for treating liver fibrosis.

The continuous progression of the aforementioned diseases increases the risk of HCC. Treatments associated with CD36 have significant value as novel therapeutic strategies for HCC. According to Tian, Li, and others, targeting pathways associated with CD36 is a promising therapeutic approach, such as targeting the Nogo-B pathway^86^ and the Cartilage oligomeric matrix protein pathway derived from HSCs.^87^ An animal study revealed that Atg5, an autophagic protein, could regulate stem cell development by modulating CD36 expression and MHC class II antigen presentation, potentially serving as a target for future antitumor therapies.^121^ In addition, TNF receptor–associated factor 3 can suppress tumor cell growth and metastasis by reducing CD36 expression and shifting macrophages from M2 to M1 polarization, representing a new approach in future cancer treatments.^122^

## CONCLUSIONS

As summarized above, CD36 has multiple roles in fat accumulation, liver inflammation, and viral replication, as well as in promoting liver fibrosis and liver cancer progression. Our review indicates a broad but underexplored research area involving the complex interplay between CD36 and the pathophysiology of liver diseases, and it summarizes current promising CD36-related therapies for liver disease. Understanding the intricate mechanisms by which CD36 contributes to liver diseases may pave the way for the development of novel therapeutic strategies. Further investigations are warranted to fully elucidate the therapeutic potential of targeting CD36 in liver diseases and to translate these findings into clinical practice.
